# From crisis to care at a refugee community health centre: a community-based participatory qualitative study of refugee experiences and outcomes

**DOI:** 10.1093/heapro/daag106

**Published:** 2026-07-28

**Authors:** Jessica Haight, Augustine Botwe, Sophie Wood, Molly Whalen-Browne, Astrid Velasquez, Tehseen Ladha, Rebecca Gokiert

**Affiliations:** School of Public Health, University of Alberta, 3-300 Dianne and Irving Kipnes Health Research Academy, 11405-87 Avenue, Edmonton, Alberta, Canada T6G 1C9; New Canadians Health Centre, B100-10615, 103 Street, Edmonton, Alberta, Canada T5H 2V6; Faculty of Education, University of Alberta, 11210 87 Avenue, Edmonton, Alberta, Canada T6G 2G5; School of Public Health, University of Alberta, 3-300 Dianne and Irving Kipnes Health Research Academy, 11405-87 Avenue, Edmonton, Alberta, Canada T6G 1C9; New Canadians Health Centre, B100-10615, 103 Street, Edmonton, Alberta, Canada T5H 2V6; Faculty of Medicine and Dentistry, University of Alberta, Walter Mackenzie Health Sciences Centre, 8440 112 Street NW, Edmonton, Alberta, Canada T6G 2R7; New Canadians Health Centre, B100-10615, 103 Street, Edmonton, Alberta, Canada T5H 2V6; New Canadians Health Centre, B100-10615, 103 Street, Edmonton, Alberta, Canada T5H 2V6; Faculty of Medicine and Dentistry, University of Alberta, Walter Mackenzie Health Sciences Centre, 8440 112 Street NW, Edmonton, Alberta, Canada T6G 2R7; School of Public Health, University of Alberta, 3-300 Dianne and Irving Kipnes Health Research Academy, 11405-87 Avenue, Edmonton, Alberta, Canada T6G 1C9

**Keywords:** refugee, principles-based care, community health centre, models of care, Canada

## Abstract

Forcibly displaced refugees often face unmet health needs and barriers to care after resettlement. In response, many resettlement countries have established specialized health clinics and centres. However, evidence on refugees’ healthcare experiences and outcomes within these diverse models of care remains limited. This study examines refugee patients’ experiences and perceived outcomes at the New Canadians Health Centre, a specialized refugee community health centre in Western Canada that provides primary healthcare during the first two years of resettlement, guided by principles for social justice, equity, and inclusion. Through community-based participatory research and qualitative description, semi-structured interviews were conducted with 31 patients, including men and women from seven different countries of birth, and analysed inductively using qualitative content analysis. Patients reported improved access to care through reduced individual and structural barriers; receipt of comprehensive care facilitated by integrated services; strong trust and cultural safety fostered by supportive patient and healthcare provider relationships; enhanced health literacy and greater capacity for system navigation; and perceived improvements in overall health and wellbeing. Opportunities to continue to strengthen care were also identified. The findings highlight key aspects of the centre’s model of care that strengthened refugees’ healthcare experiences, including phone-based interpretation, proactive patient outreach, and caring healthcare providers, along with opportunities to refine services to better address evolving needs. These insights can inform the development and enhancement of refugee-serving healthcare models across international resettlement contexts.

Contribution to Health Promotion

- Specialized refugee healthcare services designed to address unique needs meaningfully impact patient experiences and health and wellbeing outcomes.
- Targeted strategies, including proactive outreach and interpretation services, reduce individual and structural barriers to care.
- Comprehensive, integrated services, that supportive multi-disciplinary referrals, ensure patients receive appropriate clinical care.
- Patient-provider relationships built on active engagement and respect for individual cultural needs foster patient trust and safety.
- Clear information and navigation instructions strengthen system and health literacy, while promoting health outcomes.

## Introduction

Refugees flee their countries due to humanitarian crises, conflict, and persecution, and may have endured traumatic experiences, such as the loss of homes, separation from family members, and exposure to violence (Hassan *et al*. 2023). Difficult migration journeys and limited access to healthcare leave many refugees with untreated health conditions (Hassan *et al*. 2023). Studies of newly arrived refugee populations consistently document elevated rates of infectious diseases (Redditt *et al*. 2015a), substantial burdens of chronic illness (Redditt *et al*. 2015b), unmet women’s health and reproductive needs (Aptekman *et al*. 2014), and high levels of mental health concerns, such as post-traumatic stress disorder (Kirmayer *et al*. 2011). As a result, refugees require timely access to effective healthcare upon arrival in resettlement countries, which provide long-term protection following forced displacement.

Resettlement countries are responsible for providing refugees with initial support, including housing and integration services, to facilitate their transition into the new society (United Nations High Commissioner for Refugees [UNHCR] 2011). Canada is a leading resettlement country, accepting tens of thousands of refugees each year, with just under 50 000 arrivals in 2024 alone (Immigrants, Refugees, and Citizenship Canada [IRCC] 2025). In Canada, refugees are resettled through government assistance, private sponsorship, the blended visa office-referred programme that combines both, or by making an in-Canada asylum claim (IRCC 2019). Through these pathways, they receive essential support during their first year, including financial assistance, temporary accommodation, and orientation services (IRCC 2019). Healthcare services are another essential element of resettlement support internationally (UNHRC 2011). In Canada, refugees receive healthcare insurance upon arrival through the Interim Federal Health Program, which provides them with access to physicians, hospitals, and supplemental benefits until they become eligible for provincial or territorial insurance; although, co-payments were introduced in May 2026 for supplemental benefits and prescription medications (IRCC 2019). Other high-income resettlement countries also typically provide temporary health coverage to bridge initial gaps in care.

Despite healthcare coverage, international evidence shows that refugees consistently face structural barriers to accessing healthcare in their resettlement countries, including in Canada (McKeary and Newbold 2010). Refugees are unfamiliar with navigating a new system, including what healthcare services are available to them and how to access them (Coumans and Wark 2024). Health navigation is made more challenging by not speaking the language of the host country, having limited funds for transportation, and having other immediate priorities such as getting employment and finding long-term housing (Ho *et al*. 2023). Refugees may also avoid healthcare due to past traumatic experiences and a mistrust of authorities (Salami *et al*. 2020). Additionally, family physicians may be hesitant to accept refugees as patients due to their often more complex and time-intensive health challenges, unknown medical histories, and language barriers (Merritt and Pottie 2020). Canada is also facing an acute shortage of family physicians, which further limits access to primary healthcare (Duong and Vogel 2023).

Delayed or inadequate access to healthcare can exacerbate untreated conditions and increase reliance on acute care services, which can also contribute to system-level impacts such as increased use of emergency services (Thandi *et al*. 2023). In response, there is growing international recognition of the need for timely and culturally appropriate refugee healthcare services, designed in a way that effectively responds to the needs of the population. As a result, many countries have developed targeted services, often in the form of specialized refugee clinics or community health centres, to address their immediate and complex needs (Gold *et al*. 2025). In Canada, such clinics have opened in major cities, which receive large numbers of refugees, mirroring trends in the USA, Europe, and Australia (Gold *et al*. 2025).

Models of care across specialized refugee healthcare clinics vary considerably, shaped by their organizational frameworks, resource availability, and whether services are situated in primary, clinical, or community-based settings (Iqbal *et al*. 2022); however, emerging research has documented promising findings. In Canada, refugee clinics have been shown to enhance care access (McMurray *et al*. 2014) and patient reported experiences and outcomes (Reboe-Benjamin *et al*. 2023). For example, a study by McMurray *et al*. (2014) looked at care access at a refugee health clinic in Kitchener, eastern Canada, and found that after the clinic was established, wait times for refugees to see a healthcare provider upon arrival dropped by 30% and 18% more secured a permanent provider in their community after leaving the clinic. Similarly, a study by Reboe-Benjamin *et al*. (2023) explored patient experiences at a refugee health clinic in Saskatoon, central Canada, and found high patient satisfaction and self-reported improvements in health since receiving care at the clinic.

Despite these promising findings, research examining how different refugee healthcare models shape refugees’ experiences and outcomes remains limited in Canada and internationally. This is likely due, at least in part, to the challenges of conducting such studies in busy, resource-constrained clinical settings. Given the diversity of care models across countries and contexts, future research must clearly articulate the components of these models and identify which features support positive outcomes. Additionally, research should integrate refugees’ perspectives, which are essential to understand what aspects of care are most effective and responsive to their needs, but remain under-represented due to challenges accessing and engaging this population (Sulaiman-Hill and Thompson 2011). In response, this study addresses this gap in the literature by examining the impact of a refugee community health centre on healthcare experiences in Western Canada, the New Canadians Health Centre (NCHC).

The NCHC was established in 2021 in Edmonton, a metropolitan city in Western Canada, to meet the healthcare needs of newly arrived government-assisted refugees (GARs) (Haight *et al*. 2024). The NCHC provides GARs with preventative and primary healthcare, mental health services, health system navigation, and referrals to partner settlement and social service organizations during their first 2 years in Canada, before transitioning them to long-term primary care in the community. Notably, the NCHC’s services are guided by a principles-based model of care grounded in social justice, inclusion, and equity. This model integrates guiding principles throughout all aspects of healthcare delivery. Unlike conventional healthcare models that focus primarily on clinical outcomes, principles-based models emphasize both outcomes and the processes through which care is delivered, aligning care with equity-oriented values (Patton 2018).

This study builds on previous work describing how the NCHC’s model is implemented (Haight *et al*. 2026) by exploring how the model impacts refugees’ healthcare experiences and perceived outcomes. To do so, it draws directly on refugees’ first-hand accounts of their healthcare experiences at the NCHC through in-depth interviews. The study seeks to contribute to the literature by identifying aspects of healthcare that refugees themselves view as most effective for their needs.

## Materials and methods

### Setting

Established in 2021, the NCHC is located in Edmonton, Alberta, in Western Canada. The NCHC is housed within the Catholic Social Services (CSS) Reception House, which is the city’s key GAR resettlement agency. Edmonton is home to a substantial refugee population of over 50,000, accounting for 3.6% of the city’s population, which places Edmonton fifth among cities in Canada with the highest proportion of refugees per capita (Statistics Canada 2023). The NCHC serves GARs during their first 2 years in Canada, with the goal of transitioning them to a long-term primary healthcare provider after this period, to maintain capacity for new arrivals. During their time at the NCHC, GARs receive preventative and primary healthcare services; mental health services, with culturally relevant assessments, therapeutic counselling, and supportive referrals; and health navigation support and referrals to community services. These services are delivered by a team of family physicians, specialist physicians, nurses, health navigators, and operations staff (described in more detail in Haight *et al*. 2024).

Guided by a principles-based model of care, the NCHC operates according to the following foundational principles: *Social Justice, Equity, and Inclusion*, *Adopt a Social Model of Health*, *Act Collaboratively*, *Welcoming and Inclusive*, *Create a Supportive Learning Environment*, and *Community Owned*. These principles guide all aspects of the NCHC’s operations and healthcare delivery and establish standards for care. The principle of *Social Justice, Equity, and Inclusion* drives efforts to remove barriers and ensure accessible healthcare for all refugees, while also underpinning all other principles. *Adopt a Social Model of Health* promotes comprehensive support that addresses health and social care needs. *Act Collaboratively* emphasizes integrated care among health and allied professionals. *Welcoming and Inclusive* reflects the NCHC’s commitment to culturally safe (Papps and Ramsden 1996) and person-centred care (Coulter and Oldham 2016) that is responsive to their needs and recognizes patients as equal partners in care. *Create a Supportive Learning Environment* highlights a commitment to continuous learning and quality improvement. *Community Owned* underscores the active role of the community in governance and operations.

In a recent study, Haight *et al*. (2026) examined how the NCHC’s guiding principles were reflected in everyday healthcare practices, finding that all core principles were evident to varying extents across governance, service delivery, and patient engagement. Building on this work, the present study moves beyond identifying how principles are enacted in practice to examine the model’s impact on refugees’ healthcare experiences and outcomes, based on their perspectives.

### Study design

To examine the impact of the NCHC’s principles-based healthcare model, in-depth interviews were conducted with refugee patients, guided by a community-based participatory research (CBPR) approach, combined with qualitative description (Sandelowski 2000, Minkler and Wallerstein 2011). CBPR prioritizes the active engagement of communities affected by a health issue, as partners in the research process (Minkler and Wallerstein 2011), while qualitative description facilitates a rich and detailed account of a participant’s experiences (Sandelowski 2000). The inclusion of community voices was critical in this study, given that refugees are often underserved and under-represented in research.

To ensure meaningful participation, the study engaged NCHC community partners through collaboration with the NCHC’s Research and Evaluation Committee. The Committee comprises refugees with lived experience; NCHC leadership and healthcare providers, with expertise in refugee health; representatives from partner settlement agencies, specialized in refugee resettlement; and interdisciplinary researchers in public health, community health, and medicine. Committee members were involved throughout all phases of the research and their insights informed the design and implementation of culturally sensitive and contextually appropriate research strategies for refugee patients. These contributions included providing guidance on interview questions, emphasizing accessible interview options, such as in-home participation, supporting participant recruitment, and enhancing the data analysis and interpretation through member checking (described next).

### Ethical approval

Ethics approval for this research was received from the University of Alberta Research Ethics Board (No. Pro00133232). All participants signed consent forms translated into their language of preference by a language interpreter.

### Recruitment and data collection

Interviews were conducted with a total of 31 refugee patients at the NCHC, across two distinct phases that took place in August 2024 (Phase 1) and March 2025 (Phase 2). All participants were adults (18 years or older) and were currently receiving care at the NCHC or had recently transitioned out of the NCHC (within a year). Interviews were semi-structured, and participants were asked questions about the principles-based model of healthcare at the NCHC, as well as their healthcare experiences and outcomes.

Purposeful sampling was used to recruit participants based on their use of NCHC services (Suri 2011). Participants were invited due to their high level of engagement with the NCHC, either by having multiple appointments during an acute period or sustained care. Participants were also recruited with a focus on diversity in gender and country of birth, ensuring that both women and men coming from multiple countries of birth were represented. This recruitment process was facilitated by Committee members, who bridged the connection between the research team and participants. Using a list of potential participants, refugees were invited through a phone call in their primary language, with the support of student or staff interpreters. All potential participants were made aware that their involvement in research would not affect their relationships or access to services at the NCHC.

In the first phase, 26 refugees were engaged across 19 interviews (in six interviews, multiple family members participated) during August 2024. Following preliminary data analysis, it was determined that additional data collection was needed to achieve data saturation (Fusch and Ness 2015). Accordingly, in the second phase, five additional refugees were interviewed across four interviews (in one interview, two family members joined) during March 2025. Data collection and analysis occurred through an iterative process in order to confirm data saturation.

Interviews were facilitated by four graduate researchers, including the lead author (J.H.), with the support of language interpreters. All interviews but one was conducted in refugees’ primary languages other than English, including Arabic, Pashto, Dari, Kinyarwanda, Somali, Swahili, and Spanish. The trained language interpreters played a key role in ensuring that patients understood the interview questions by rephrasing them as needed. Interviews were conducted in person where the participant felt most comfortable; at the NCHC, in public settings, or at home, which helped to mitigate transportation and childcare costs. Of the 23 interviews, 16 took place in person at the NCHC, six in homes, and one at a local public library. Interviews were audio-recorded using a hand-held recorder and lasted an average of 36 min, with a range from 14 to 80 min. Refugee participants received a $20 gift card for their time and participation.

### Data analysis

The interviews were translated and transcribed verbatim by the trained interpreters, while the English interview was transcribed using Otter.ai. Since data collection took place across two distinct stages, interviews from each stage were first analysed separately. The two datasets were then compared to identify points of convergence and divergence, and showing conceptual similarity, they were subsequently synthesized. The complete dataset was analysed using Hsieh and Shannon’s (2005) approach to conventional qualitative content analysis. Analytical questions guided the process, including the following: Do patients report better access to healthcare? Do patients receive timely and appropriate medical treatment? Are patients satisfied with care and providers? Do patients feel like providers are respectful and supportive? Do the NCHC’s providers address patients’ needs? Do patients increase their health and navigation knowledge? Do patients report improvements in health outcomes? Guided by these questions, J.H. identified codes and organized them into categories. The emerging findings were then discussed amongst a team of researchers to verify interpretations (J.H., A.B., S.W., and R.G.). Member checking was also conducted to verify findings with three NCHC staff members who are also Committee members and co-authors on this study (M.W.B., A.V., and T.L.). This checking process focused on verifying accuracy and providing contextual clarification, including details related to the NCHC’s model of care and service delivery. This feedback enhanced the rigour of the study but did not alter the presentation of refugees’ experiences or interpretation of findings.

### Positionality

The authorship team included J.H., lead author and doctoral candidate specializing in community-engaged health systems research and evaluation; A.B., doctoral candidate specializing in mixed-methods research, evaluation, and early childhood development; S.W., post-doctoral fellow focused on youth mental health; M.W.B., family physician, the NCHC’s Medical Director, and Assistant Clinical Professor; A.V., Certified Resettlement Practitioner and the NCHC’s Executive Director with lived experience as a refugee; T.L., paediatrician and Assistant Professor specializing in health equity; and R.G., Professor with decades of experience in community-engaged research and evaluation. Together, the team brings diverse perspectives across research, evaluation, practice, clinical care, and newcomer lived experience, which enhanced this work by integrating diverse insights and adding important context to the interpretation and presentation of findings.

## Results

In-depth interviews took place with 31 refugee patients at the NCHC, including 18 women and 13 men. Patients came from Afghanistan (*n* = 12), Syria (*n* = 5), Sudan (*n* = 5), the Democratic Republic of the Congo (*n* = 5), Iraq (*n* = 2), Somalia (*n* = 1), and Venezuela (*n* = 1). These patients provided valuable insights into the NCHC model’s impact on their healthcare experiences and outcomes. The findings are organized into five overarching themes, which are summarized in Table 1 and described below. Patient quotes are integrated for illustration purposes. Additionally, the narrative includes the number of patients who raised specific points within the themes to provide context; however, the absence of a comment should not be interpreted as a lack of a shared sentiment. Many conversations naturally focused on different aspects of the patient experience, and certain sentiments may not have surfaced simply because they were not the central topic of discussion.

**Table 1:** Overview of patients’ healthcare experiences and outcomes.

Theme and description	Illustrative quote
**Increased healthcare access through reduced individual and structural barriers**	
Patients reported improved access to healthcare through reduced individual and structural barriers. Phone-based interpretation helped to address language barriers, targeted transportation support made access easier, and proactive patient outreach through reminders and follow-ups kept patients informed about care.	*‘They monitored us all the time. Now, everything is better…We book fewer appointments now. We're happy with everything’.*
**Appropriate healthcare enabled by comprehensive services and referrals**	
Patients reported appropriate healthcare that addressed their needs. Comprehensive medical screening identified health concerns, while holistic services and referrals addressed diverse needs. However, some patients noted the need for shorter wait times and increased physician availability.	*‘The medical services…there wasn’t anything missing…everything I’ve come here for I’ve found here’.*
**Trust and cultural safety through supportive patient-provider relationships**	
Patients reported supportive interactions with healthcare providers, who are welcoming and inclusive, take time to listen to patients’ concerns, engage them in treatment, and are respectful and accommodating of patients’ cultural and individual preferences, making patients feel safe to share concerns and engage in their care.	*‘When we first arrived, we felt love, care, everything. There was a big restfulness…We felt safety and peace’.*
**Capacity building through improved system and health literacy**	
Patients reported improved capacity in healthcare navigation through support delivered in partnership with a settlement partner. Health literacy is enhanced by healthcare providers sharing in-depth and clear information about health and addressing questions and concerns.	*‘When you come as a new person. You don’t know anything…but you come and learn a lot of things about your own self, your own body’.*
**Perceived improvements and positive changes in health and wellbeing**	
Patients reported noticeable improvements in their health after receiving care at the NCHC. This included diagnoses, appropriate treatment, and access to necessary medications, referrals, and services. Many refugees described being mentally and physically healthier because of the care.	‘*I cannot talk enough about what they did for me. They improved and helped me a lot’.*

### Increased healthcare access through reduced individual and structural barriers

Patients said that the NCHC made it easier to access healthcare by reducing common barriers that they face, proactively reaching out to them, and supporting them with individual needs. The NCHC provides a phone-based language interpretation service, which enables patients to communicate with healthcare providers in their primary language, as noted by Patient 4: ‘*They will bring this mobile thing…The telephone. They call, and we converse’.* In some cases, patients face challenges with this phone service due to translation inaccuracies and regional differences in dialect, including a patient who struggled with linguistic differences in Swahili: ‘*Communication was a challenge…because the Swahili spoken in Tanzania and Congo is very different’* (Patient 10). Some patients also expressed a preference for in-person interpreters rather than translation over the phone, which is occasionally accommodated at the NCHC by team members who speak multiple languages, such as Arabic, as noted by Patient 4: ‘*There are people who are employed here who speak Arabic…they provided assistance’.* Recognizing the limitations, patients described the NCHC’s phone-based interpretation as significantly improving their access to care (mentioned by 24 patients): ‘*The translator talks to us, and this allows us to understand’* (Patient 7).

Patients also said that the NCHC addresses transportation barriers when they first arrive in Canada, reducing challenges with navigation. Upon arrival, many patients are housed in the CSS Reception House during their first weeks in Canada, co-located in the same building as the NCHC. Patients explained that this co-location model makes it easy for them to access initial appointments, as noted by Patient 5: ‘*They [NCHC team members] would take us and bring us downstairs…in case we need drugs, we need needle[s], or we need to do an examination’.* For patients not initially hosted in the CSS Reception House, the NCHC coordinates with CSS on transportation to initial appointments, either by supporting public transit or arranging taxi services: ‘*They come pick you up, take [you] to their clinic’* (Patient 27) (mentioned by 6 patients). For patients facing mobility constraints or exceptional circumstances, they continued to receive this support on a case-by-case basis.

Furthermore, patients said that the NCHC supports them through proactive outreach, including appointment reminders and regular follow-up or check-in calls. Patient 10 explained: ‘*They would regularly call to check on me and remind me of appointments’.* While the NCHC routinely provides patients with reminders of upcoming appointments, check-in calls are based on individual patient needs, particularly for those undergoing periods of significant life challenges. Patient 10, who experienced challenging periods of time, further described this, ‘*[They] were like family to me during those difficult times…They checked on me often. They made me feel like we were together through it all’.* Not all patients reported receiving this type of proactive engagement, with Patient 13 sharing: ‘*What I would suggest is that they should have someone check on you regularly, maybe monthly, to see how you’re doing’.* However, other patients felt well-supported through frequent outreach (mentioned by 7 patients), as noted by Patient 21: *‘They monitored us all the time. Now, everything is better…We book fewer appointments now. We're happy with everything’.*

### Appropriate care enabled by comprehensive services and referrals

Patients said that the NCHC provides appropriate healthcare that addresses their needs. This starts with a comprehensive medical screening upon their intake (mentioned by 6 patients). Patient 28 described receiving screening: ‘*I do not think there was anything left out…There was no place on my body that was not tested, from eyes, stomach, kidney, and tonsils…Everywhere and everything’.* Similarly, Patient 27 shared: ‘*They did all blood tests, they tested my stomach, they did an X-ray and everything’.*

In addition to screening, patients emphasized that the NCHC’s holistic range of healthcare services (clinical services, mental health services, and health navigation support) addresses their diverse health needs (mentioned by 15 patients). Patient 30 explained, ‘*Anything you would need, they would provide it for you’.* Patient 10 also emphasized: ‘*The medical services…there wasn’t anything missing…everything I’ve come here for I’ve found here’.*

Most patients also said they can readily access appointments at the NCHC without delays, as Patient 11 described: ‘*I did not have any problem. When we needed to see a doctor, we called…and they booked an appointment for us’.* Alternatively, some felt like there were long wait times for appointments (mentioned by 9 patients), as noted by Patient 11: ‘*In case of an emergency, it took about 10 to 15 days to see a doctor. If it was not an emergency case, it took between 25 days to 1 month’.* In such cases, patients recognized that wait times can be attributed to the high volume of refugees seen at the NCHC, as noted by Patient 27: ‘*The line [of] people may be a lot…there is a lot of refugees, right? So just maybe you wait for a time’.* In response, patients emphasized the need for more healthcare providers to keep up with needs, as further noted by Patient 28: ‘*They can…have more staff, because they don’t have a lot of doctors’.* Additionally, patients said that they are often seen by different healthcare providers for each appointment at the NCHC, depending on who is available each day. Some patients found the switching between providers to be challenging ‘*because every time you were repeating all the information that you had’* (Patient 29). Alternatively, other patients were unaffected by changes in providers, as described by Patient 31, ‘*For me, I had no issue with that; what really mattered for me is my problems and health issues be handled and solved properly’.*

When patients require specialist or allied healthcare services beyond the scope of the NCHC, they said that they are promptly referred to these services, as noted by Patient 27: ‘*What I was not able to get from them [NCHC], that’s what they would refer me…and I see a specialist…they know that you will go and see a solution’.* In some cases, patients expressed a preference for receiving all services directly at the NCHC to avoid the challenges of navigating external referrals; however, they acknowledged the limitations in what the NCHC can provide, as noted by Patient 4: ‘*If all services are in one place it would be better, but [the] ophthalmologist is outside [the NCHC], dentists are outside, and the X-ray’.*

### Trust and cultural safety through supportive patient-provider relationships

Patients consistently described meaningful and positive interactions and relationships with healthcare providers at the NCHC, which promotes a sense of trust and cultural safety. They said that providers at the NCHC are welcoming and inclusive (mentioned by 26 patients), as described by Patient 7, ‘*When we come here, they [NCHC team members] all know us. They treat us as if we are their relatives’.* Patient 30 described their meaningful experience of first arriving at the NCHC: *‘When we first arrived, we felt love, care, everything. There was a big restfulness...We felt safety and peace’.*

In addition to being welcoming, patients said that NCHC providers take the time to carefully listen to their concerns and actively elicit their input in their treatment (mentioned by 24 patients). This was described by Patient 31, who arrived in Canada as a single mother, ‘*When I arrived here [at the NCHC], I saw everything is okay because they listen to you…I am so happy seeing someone like me, a single mother, and they listen to you very well’.* Patient 12 also noted, ‘*They ask about your illness and won’t give you medication without hearing what you have to say’.* For some patients, being actively engaged in their care in this way marked a distinct difference from their previous experiences. Patient 13 further explained that in their country of origin, ‘*You’re used to just waiting for the doctor to tell you what’s going to happen…You don’t usually get to decide or question things’.* Patient 21 shared their reflections on how care at the NCHC differed from experiences at other clinics in Canada:

There's a lot of difference between a family doctor outside and a family doctor at the NCHC…[At the NCHC] they listen very carefully to the patient. They listen to the patient's story. Family doctors [outside of the NCHC] don't listen to the patient much. [Here], the patient is happy that he is heard, and they'll get a good result.

Patients also said that providers are respectful and accommodating towards their cultural and individual backgrounds and preferences (mentioned by 12 patients). Patient 27 explained that providers at the NCHC ‘*are people that have been coach[ed] [about] cultural differences and how to respect [people]’.* Patient 27 further noted, *‘They respect all the culture[s] of people that come into the institution’.*

Altogether, when healthcare providers are welcoming, listen to and engage patients in care, and are respectful, patients said they felt comfortable sharing their health concerns, as noted by Patient 12: ‘*Once you see a doctor smiling and is behaving very kindly, you feel safe to talk about your problem’.* Patient 27 shared, *‘When you meet another human being who is there to help you, even you will let your guard down…you will be open and be able to share that human being’.* Patients said that they feel trust and safety at the NCHC, enhancing their experience of care, as Patient 31 noted: ‘*I received the best service…I felt I was in safe hands’.*

### Capacity building through improved system and health literacy

Patients shared that the NCHC supported them in strengthening their health literacy, through coordinated efforts with CSS. Early on, patients frequently struggle to navigate the healthcare system outside of the NCHC, for which the NCHC provides support in arranging rides to initial appointments in collaboration with CSS, as described by Patient 7: ‘*One day…we had to go to another hospital for the blood test. They looked after us here and provided…food for lunch and also gave us a ride’.* The NCHC also refers patients to the CSS for help with navigating healthcare systems. Patient 28 explained that he had someone from CSS to ‘*show me the city buses, how to ride, how to ride the train’.* Patients described feeling more equipped to navigate healthcare appointments outside of the NCHC, as described by Patient 28: *‘I didn’t know a clinic or place [to go to], and I didn’t even know what to say. They [the NCHC] used to hold my hand…Now I am good’.* However, one patient noted that navigation instruction could be more comprehensive.

In addition, patients said they receive in-depth health information. Specifically, providers share detailed information with patients about ‘*the illness, about the medicine, how to use it, when to use it’* (Patient 9). Patient 20 emphasized: ‘*[They] would give us a lot of advice’.* However, one patient noted that they would like more detail from their provider on the results of their blood test: ‘*They only did blood work once and didn’t discuss the results’.* In contrast, most patients said that information was presented in ways that were clear and helped to improve their understanding (mentioned by 14 patients), as noted by Patient 5: ‘*The communication is always very good’.* In particular, when patients had concerns about their treatment plan or required additional clarity, they said that providers were able to adequately address their concerns. This information, in turn, helped patients to learn more about their health, as noted by Patient 27: ‘*I have learned a lot about my health’.* Patient 27 further shared: ‘*[NCHC providers] are like [a] first responder, when you come as a new person. You don’t know anything…but you come and learn a lot of things about your own self, your own body’.*

### Perceived improvements and positive changes in health and wellbeing

Finally, patients reported perceived improvements and positive changes in their health since receiving care at the NCHC. For many patients, previously undiagnosed or untreated problems were discovered and addressed at the NCHC. For example, Patient 21 discussed receiving treatment for his wife: ‘*My wife had a problem with tuberculosis in the past. They diagnosed that she still had symptoms of tuberculosis. They gave her medicine’.* Patient 19 shared about learning of a vitamin deficiency: ‘*They called us and told us there’s a certain deficiency and sent us medication. We used them, later they ran tests again and told us we are okay’.* Additionally, Patient 5 discussed learning that his children have food allergies:

They helped us with the issues that we did not understand and did not know about. For example…they advised on what type of food the child may have allergies to. They [had a] very important role in the development of the child.

Most patients expressed relief upon receiving care; however, in one instance, a patient attributed their child’s new diagnoses to the NCHC itself, rather than recognizing them as previously unidentified conditions, underscoring the importance of communication about diagnoses and treatment. Specifically, Patient 2 shared: ‘*[Name of child] did not have any serious problems but when they came here [the NCHC], he started having two problems’.*

While a couple of patients said that they have yet to see major health improvements, many patients reported that their health improved since receiving care at the NCHC (mentioned by 17 patients). For example, Patient 25 said that her child experiences less leg pain since receiving treatment: ‘*My daughter constantly had cramps in her legs. When we came, they prescribed her medication, and it has been four months since she has gotten a cramp’.* Similarly, Patient 1 shared that the ‘*NCHC helped my son to become healthier through…prescribing vitamins’* and that without the NCHC, ‘*I think his health wouldn’t be great like now’.* Additionally, for Patient 28, the NCHC helped address his son’s needs:

I had a young disabled [child]…They did multiple and a lot of tests including scan…They booked an appointment for an operation…One year later, the appointment came. The child is very good now. I cannot talk enough about what they did for me.

Finally, patients not only spoke about gains in physical health, but also about how support affected their mental health and wellbeing. For example, Patient 30 described how his life experiences have affected his mental health: ‘*I have suffered a lot, honestly…I have gone through and lost hope and felt a lot psychologically’.* However, after receiving care at the NCHC, he went on to share that the kind and supportive staff made a key difference in his life: *‘It could possibly be one person who makes you overcome this crisis that you are feeling…through a smile, through a sweet word, through listening, through interacting with you’.* Patient 11 also shared the meaningful impact of the centre: *‘Before I went to the center, I had a lot of problems…I went there [to the NCHC] and I was very happy, and I felt secure. I found I can support myself’.*

## Discussion

The first-hand perspectives of refugees provided rich insights into their healthcare experiences and perceived outcomes, addressing a notable gap in the literature on healthcare effectiveness from refugees’ perspectives. Patients identified several aspects of the NCHC’s model of care that contributed to positive impacts within the context of large-scale systemic healthcare barriers, along with opportunities for further strengthening the model.

Patients at the NCHC emphasized its role in reducing barriers and improving care access, most notably through the provision of phone-based interpretation services. Refugees consistently highlighted that communicating in their primary language made it easier to engage and meaningfully benefit from care. Literature reinforces the importance of interpretation for accurate medical communication and treatment adherence, along with patient satisfaction (Flores 2005). For example, Reboe-Benjamin *et al*.’s (2023) study of the refugee clinic in Saskatoon also found that refugees valued interpretation services, with over 94% (*n* = 61/65) of refugees reporting that interpretation was helpful. While some patients at the NCHC expressed a preference for in-person interpretation, and other studies have shown its link with improved satisfaction (McBride *et al*. 2017), in-person interpretation can be cost prohibitive. More recently, artificial intelligence-based translation tools have begun to emerge, with the promise of being cost-effective; however, many struggle with different dialects, particularly non-European languages (Genovese *et al*. 2024), constraining their current utility for refugee health.

Other aspects of care, including transportation support and active patient outreach, were also noted by NCHC patients to facilitate easy access and engagement in care. The NCHC collaborates with the CSS on transportation support on a case-by-case basis. Patients who received this support appreciated its role in making care easier to access, particularly upon their first few months in Canada or if they faced mobility constraints. Transportation supports have also been adopted by some other refugee healthcare models, particularly in Australia (Joshi *et al*. 2013), including an integrated refugee healthcare service in Melbourne, Australia (McBride *et al*. 2017). However, because transportation programmes can be resource-intensive, they are challenging to implement broadly, unless delivered in partnership with settlement services or medical transportation programmes. At the NCHC, many newly arrived refugees are temporarily housed in the CSS Reception House during their first few weeks in Canada, eliminating transportation needs during that early period and facilitating easy access to care.

Patients at the NCHC described receiving comprehensive healthcare that meets their needs, including medical screening, interdisciplinary treatment, and appropriate referrals. Patients emphasized that the NCHC was able to provide or coordinate the care they required, although many also noted that long wait times can be a challenge. The NCHC aims to see newly arrived patients within one to three months of arrival, and wait times are contributed to by limitations in staff capacity relative to the needs of patients. Patients were often understanding of the reason for delays. Such challenges with wait times are not unique to the NCHC; instead, they are more broadly reflected across Canada, where a family physician shortage places a strain on the primary healthcare system (Li *et al*. 2023). It remains unclear whether wait times for newly arrived refugees would be different in the wider community; however, as noted previously, the study by McMurray *et al*. (2014) suggests that dedicated refugee health clinics may help reduce initial delays by 30%, improving access to care.

Refugees repeatedly expressed appreciation for the NCHC’s healthcare providers, describing them as inclusive, welcoming, and supportive. Patients reported that providers took the time to listen to their concerns and elicit their perspectives in treatment, and that they were respectful and accommodating towards their cultural and individual preferences. Patients said that this supportive care made them feel comfortable in sharing their health concerns, a pattern also supported in literature showing that positive patient-provider relationships facilitate increased symptom sharing, treatment engagement, and patient satisfaction (Beach *et al*. 2005). Patient experiences at the NCHC also demonstrate that person-centred care and culturally safe practice (Papps and Ramsden 1996, Coulter and Oldham 2016) are not simply principles at the centre, but actively and meaningfully lived by patients, whose perspectives offer the most credible mechanism for assessing care quality.

Notably, patients at the NCHC emphasized feelings of safety, belonging, and support from healthcare providers more frequently than they discussed the extent to which the NCHC addressed their medical needs, underscoring the meaningful impact of relational aspects of care. This is significant because supportive patient-provider relationships may not only enhance patient satisfaction, as found in other refugee healthcare studies (McBride *et al*. 2017), but may also have indirect influences on overall wellbeing (Aubé *et al*. 2019). For example, Aubé *et al*. (2019) found that supportive relationships between migrant mothers and providers at a perinatal health centre in Montréal, eastern Canada, played a key role in strengthening resilience among newcomer mothers.

Patients at the NCHC also reported that their knowledge about their health, as well as their understanding of the broader healthcare system, increased at the NCHC. Some patients described health navigation support delivered by CSS, as beneficial, which is also mentioned in literature as a tool for building a patient's self-confidence, autonomy, and reducing inefficient system use (Teggart *et al*. 2023). Enhanced personal navigation skills are particularly important when individuals face additional system-level barriers, such as language barriers, which can place increased responsibility on patients to navigate care. However, one patient noted that teaching about navigation could be made more robust, indicating an opportunity to strengthen efforts. Any effort to enhance health navigation will need to balance limited capacity and resources. Online tools and programmes have been increasingly introduced and show promise to facilitate and streamline health navigation learning, such as the HEalthCare NAvigation Competency Scale in the USA (Yeo *et al*. 2025), which could be explored for its application and use in Canadian contexts.

In contrast, more NCHC patients emphasized that their understanding of their personal health improved as a result of clear, detailed, and accessible information. For some refugees, they had not previously had access to comprehensive healthcare or knowledge about how to care for their health; therefore, these experiences at the NCHC reflected important opportunities to improve health literacy. Research shows that health literacy plays a critical role in strengthening patients’ ability to participate meaningfully in care decisions, self-advocate, and take care of their health (Ishikawa and Yano 2008). Other studies have shown similar findings in the role of refugee health services in supporting health literacy, including McBride *et al*.’s (2017) evaluation of the integrated health services in Melbourne, which found that 83% (*n* = 131/159) of patients agreed that they understood the health explanations provided and learned more about managing their own health. While improving healthcare literacy is not a direct goal of the NCHC, it is likely facilitated through health navigation support, as well as healthcare providers taking the time to answer questions and actively engaging refugees in their care.

Finally, patients self-reported noticeable improvements in their health. They described gaining a better understanding of their diagnoses and experiencing improvements in their health through treatment provided at the NCHC or through referrals to specialists or allied healthcare. These findings align with those of Reboe-Benjamin *et al*. (2023), who found that 57% (*n* = 41/72) of respondents at the refugee clinic in Saskatoon reported improved health, and McBride *et al*.’s (2017), who found that 83% (*n* = 131/159) patients at the health services in Melbourne indicated that their health concerns were adequately addressed. Future studies could strengthen these conclusions by verifying improvements through health record data. However, refugees perceived meaningful improvements in their health, which indicates that principles-based healthcare provided through the NCHC positively impacts refugee health.

### Limitations

This study had strengths and limitations. A key strength was the large sample of refugees, an often hard-to-reach and under-represented population in research. Accessibility was supported through interviews in patients’ primary languages with interpreters. While the researchers and language interpreters aimed to clarify meaning when uncertain, the translation process still may have missed cultural nuances, and the transcription process may have introduced additional errors, potentially misrepresenting a patients’ original message (Choi *et al*. 2012). Another limitation is that most patients had multiple visits at the NCHC over an acute period or received care for a sustained period, potentially giving them more time to establish relationships and hold positive viewpoints. A future study would benefit from including refugees with fewer appointments to capture diverse viewpoints. Additionally, although patients were informed in their primary languages that honest feedback would not affect their relationship with the NCHC, some may still have hesitant to voice criticism. This may have been mitigated by interviewing both current and former patients, as they may feel more able to speak freely. Finally, as findings are based on the patient’s self-reported viewpoints, future research could further strengthen the results by pairing their perceived healthcare engagement and outcomes with health record data over time.

## Conclusion

Refugee patients provided key insights into the impact of principles-based healthcare and elements of responsive refugee healthcare. At the NCHC, patients reported improved access through reduced individual and structural barriers; receipt of appropriate and comprehensive care facilitated by integrated services and timely referrals; strong trust and cultural safety fostered by supportive patient and healthcare provider relationships; greater capacity to navigate the health system and enhanced health literacy; and perceived improvements in their overall health and wellbeing. They also identified opportunities to improve wait times and provide more robust health navigation support. These findings demonstrate the importance of language interpretation, patient-centred and culturally safe healthcare approaches, and comprehensive support for meeting the needs of refugees. These insights can inform the development and enhancement of refugee-serving healthcare models in Canada and internationally.

## Data Availability

The data underlying this study cannot be shared publicly to protect the privacy of participants. Inquiries regarding the data can be made to JH, jbhaight@ualberta.ca.
